# Early detection of left atrial and bi-ventricular myocardial strain abnormalities by MRI feature tracking in normotensive or hypertensive T2DM patients with preserved LV function

**DOI:** 10.1186/s12872-020-01469-2

**Published:** 2020-04-23

**Authors:** Guozhu Shao, Yukun Cao, Yue Cui, Xiaoyu Han, Jia Liu, Yumin Li, Na Li, Tong Liu, Jie Yu, Heshui Shi

**Affiliations:** 1 Department of Radiology, Union Hospital, Tongji Medical College, Huazhong University of Science and Technology, Wuhan, 430022 China; 2 Hubei Province Key Laboratory of Molecular Imaging, Wuhan, 430022 P.R. China

**Keywords:** Diabetic cardiomyopathy, Hypertension, Magnetic resonance imaging, Feature tracking, Strain

## Abstract

**Background:**

Previous studies have found that impaired global myocardial systolic strain is associated with cardiovascular events in T2DM patients. However, the effect of hypertension (HT) on left atrial (LA), right ventricular (RV) and left ventricular (LV) myocardial deformation in hypertensive T2DM patients has not been fully studied by cardiac magnetic resonance feature tracking (CMR-FT). Our aim was to assess LA, RV and LV strain in T2DM patients with T2DM-HT and without hypertension using CMR-FT and to determine the underlying relationships with clinical parameters.

**Methods:**

A total of 27 T2DM patients, 23 T2DM-HT patients and 31 controls were studied. LA, LV and RV strain was evaluated using CMR-FT. The clinical and biochemical parameters of the patients were collected.

**Results:**

The T2DM patients had reduced LA global circumferential strain (LAGCS), radial strain (LAGRS), longitudinal strain (LAGLS) and right ventricular longitudinal strain (RVGLS) compared with the controls (LAGCS: 27.2 ± 2.1% vs 33.5 ± 2.4%; LAGRS: − 28.6 ± 1.1% vs − 31.9 ± 1.3%; LAGLS: 24.3 ± 1.3% vs 31.4 ± 1.5; RVGLS: − 21.4 ± 1.2% vs − 26.3 ± 1.1%, *p* < 0.05 for all). The T2DM-HT patients had greater LAGCS, LAGRS and LAGLS than the T2DM patients (LAGCS: 40.4 ± 3.8% vs 27.2 ± 2.1%; LAGRS: − 36.8 ± 2.0% vs − 28.6 ± 1.1%; LAGLS: 32.3 ± 2.4% vs 24.3 ± 1.3%, *p* < 0.05 for all). In the diabetic patients, LAGCS was associated with microalbuminuria levels (standardized ß = − 0.289, *p* = 0.021), and LAGCS, LAGRS and LAGLS were correlated with diuretic treatment (standardized ß =0.440, − 0.442, and 0.643, *p* < 0.05 for all).

**Conclusions:**

CMR-FT may be considered a promising tool for the early detection of abnormal LA and RV myocardial strain. LA and RV strain values are impaired in T2DM patients. The amelioration of LA strain might be associated with hypertensive compensation or antihypertensive treatment, which requires to be confirmed in larger trials.

## Background

Cardiovascular disease (CVD) is the key cause of morbidity and mortality in patients with type 2 diabetes mellitus (T2DM) [1]. Diabetic cardiomyopathy (DCM) is defined as abnormal cardiac structure and function that is independent of coronary artery disease (CAD) and hypertension and can lead to heart failure [2]. Hypertension (HT) is a common concomitant condition in the majority of T2DM patients, and its coexistence contributes to a four-fold increased risk of cardiovascular mortality compared with normal controls [3]. In addition, these two conditions are associated with structural and functional atrioventricular abnormalities. Consequently, in cases of left atrial (LA), left ventricular (LV) and right ventricular (RV) structure and function, early quantitative detection and timely intervention are crucial to the management of normotensive or hypertensive T2DM patients.

At present, cardiac MRI (CMR) is the most accurate noninvasive method for evaluating cardiac structure and function, although it has lower temporal resolution than echocardiography does [4]. Numerous studies on CMR imaging in T2DM patients have mainly focused on LV [5, 6]. Recently, a novel technique, cardiac magnetic resonance feature-tracking (CMR-FT), has been regarded as a more sensitive tool for measuring myocardial deformation as an indicator of subclinical myocardial dysfunction [7]. CMR-FT has been increasingly used for myocardial strain evaluation in various types of cardiomyopathy, such as cardiac amyloidosis [8], hypertrophic cardiomyopathy [9], and dilated cardiomyopathy [10], and the reproducibility of CMR-FT has been well demonstrated [11]. However, analyses of the role of LA and RV deformation in DCM by CMR-FT, especially with coexisting hypertension, have rarely been reported.

The identification of early systolic function derangements can be achieved with the use of myocardial deformation [12]. Consequently, the purpose of this study was to quantify MRI-derived LA, LV and RV strain alterations in normotensive or hypertensive T2DM patients and to investigate their association with clinical indicators.

## Methods

### Study population

From July 2017 to April 2018, 52 consecutive patients (27 T2DM patients and 25 T2DM-HT patients) were retrospectively recruited from the Department of Endocrinology at Wuhan Union Hospital. Additionally, 32 healthy volunteers matched for age, sex and BMI were recruited from the local population and served as control group. HT is defined as a history of hypertension or treatment with antihypertensive drugs or continuous blood pressure (BP) measurement > 140/90 mmHg [13]. For inclusion, T2DM patients were required to meet the World Health Organization standards [14]: age 30–70 years, no history of heart disease, and a normal physical examination and ECG. The inclusion criteria for the controls were no history of hyperlipidemia, hypertension, diabetes mellitus, or cardiovascular, peripheral vascular or cerebrovascular disease; normal findings on routine physical examination, including a normal ECG and echocardiogram; and no use of any cardioactive medications. The exclusion criteria included clinical evidence of coronary artery disease, myocardial infarction, dilated cardiomyopathy, valvular heart disease, renal failure (glomerular filtration rate (eGFR) < 30 ml/min), contraindications to MR imaging, the presence of abnormal cardiac dimensions and abnormal wall motion and cardiac insufficiency (LV ejection fraction (LVEF) < 50%). All subjects signed written informed consent forms in this study, which was approved by the ethics committee of our institution.

### Anthropometric and biochemistry evaluations

The sex, age, height, body weight, and BP of all subjects were collected. Blood samples were obtained under fasting conditions before the MRI examination. Laboratory tests, including tests for glycosylated hemoglobin (HbA1c), microalbuminuria (MA), medications, serum glucose, fasting blood samples, creatinine, triglycerides (TG), total cholesterol (TC), blood urea nitrogen (BUN), high-density lipoprotein cholesterol (HDL-C) and low-density lipoprotein cholesterol (LDL-C) were performed for all patients within 1 week of this study. Low-density likpoprotein (LDL) cholesterol was calculated according to the Friedewald equation.

### CMR scanning protocol

All subjects underwent a standard CMR examination with a 1.5 T scanner (MAGNETOM Aera, Siemens Healthcare, Erlangen, Germany). A balanced steady-state free procession (b-SSFP) sequence was used to obtain cine images, including the acquisition of three long-axis (two-, three-, and four-chamber) and short-axis (coverage from the base to the apex segment) slices. The cine image parameters were as follows: repetition time (TR)/echo time (TE), 2.9/1.2 ms; slice thickness, 6 mm; flip angle, 80°; FOV, 360 × 270 mm^2^; matrix, 144 × 256 pixels; voxel size, 1.3 × 1.3 × 8.0 mm^3^; and scanning time, the duration of 11 heartbeats.

### Assessment of cardiac volume and function

Argus software (Syngo MMWP VE30A workstation, Siemens) was used to analyze cardiac structure and function. The LV function parameters, LV end-diastolic (LVEDV), end-systolic volume (LVESV), stroke volume (SV), ejection fraction (LVEF) and mass (LVM) were measured by manually tracing the endocardial and epicardial contours on all contiguous short-axis cine images. Furthermore, the LA volume (LAV) was calculated using the biplane area-length method (LAV = [0.85 × (2-chamber area) × (4-chamber area)]/L, where L was the shortest dimension from the back wall to the line across the hinge points of the mitral valve between the above two chambers above it). The LA appendage and the pulmonary veins were excluded from the measurements. All parameters were indexed to the body surface area.

### CMR feature tracking analysis

**Fig. 1:**
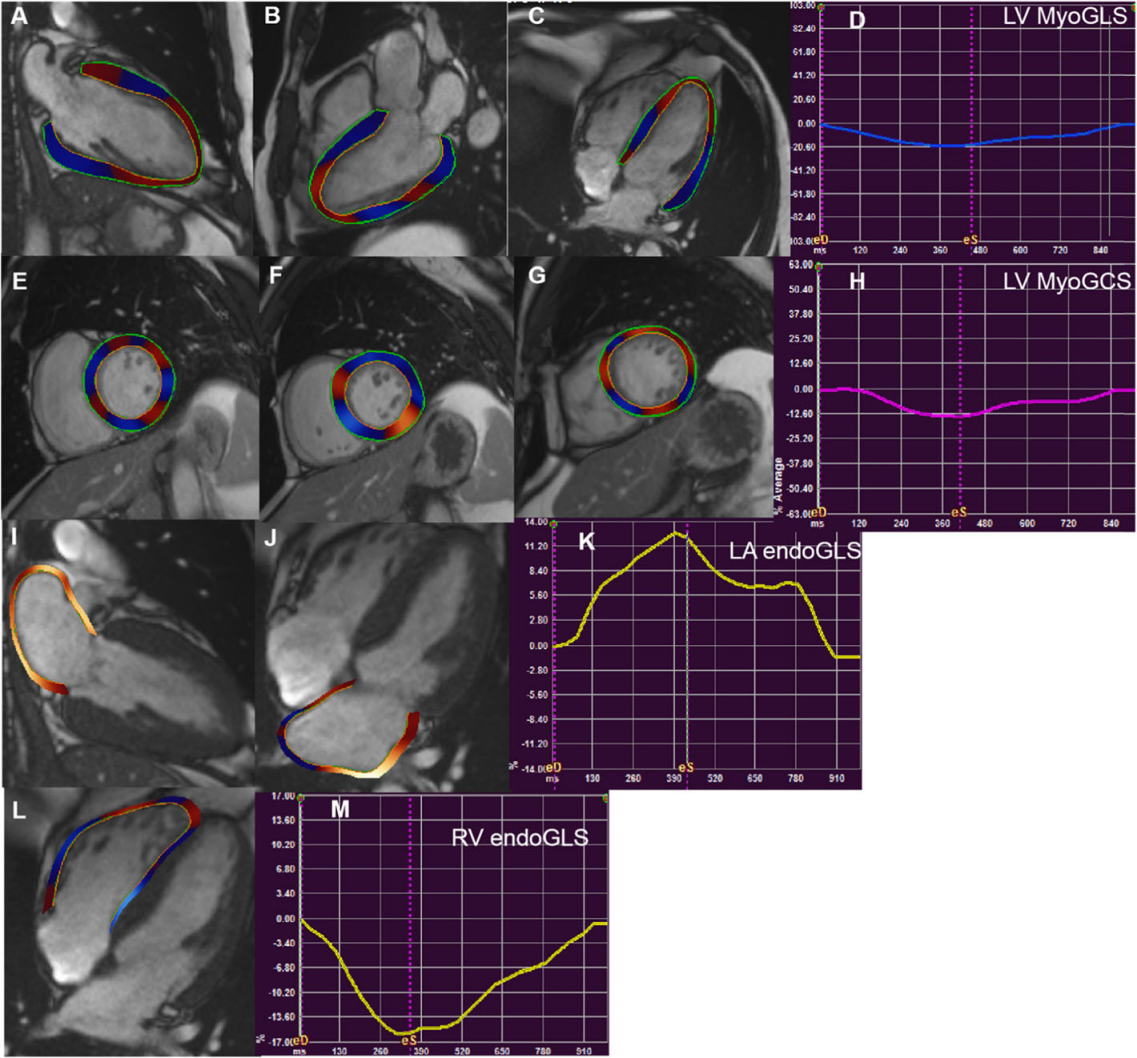
Representative images of a healthy volunteer in long axis (**a–c**) and short axis (**e–g**) directions and strain curves. Representative contour of the endocardium and endocardium of the left ventricular in the 2-, 3-, 4-chamber (**a、b、c**) and corresponding LV MyoGLS (**d**). Endocardial and epicardial borders in base, mid, apical short axis views were presented (**e-g**) and corresponding LV MyoGCS (**h**). Left atrial endocardial boundaries were represented in 2-, and 4-chamber (**i-j**) corresponding LA endoGLS (**k**). Right ventricular endocardial boundaries were represented in 4-chamber (**l**) and corresponding RV endoGLS (**m**)

Data were analyzed and processed using commercial cardiovascular postprocessing software (Medis 3.0, Netherlands) to obtain global measurements of LA, LV and RV strain. Two-, three-, and four-chamber long-axis images were imported into the software. At the end of diastole, the left ventricular endocardial and epicardial contours were manually delineated on the short axis and the long axis, respectively. The trabecular and papillary muscles were included within the endocardium **(**Fig. 1a–c, e–g**).** The LA endocardium was delineated in the 2-and 4-chamber views of the LA **(**Fig. 1i, j**)**. The endocardium of the RV was outlined in 4-chamber view (Fig. 1l). LV global longitudinal strain (LVGLS), LV global circumferential strain (LVGCS), left atrial global radial strain (LAGRS) and right ventricular global longitudinal strain (RVGLS) were automatically extracted from the corresponding strain curves **(**Fig. 1d, h, k, and m**)**.

LAGLS, left atrial global circumferential strain (LAGCS), and left atrial global circumferential strain (LAGRS) were measured in both two- and four-chamber views, although pulmonary vein confluence and LA appendages were not included. The LVGRS and LVGCS were measured in the short axial field of view. LVGLS measurements were obtained in 2-, 3-, 4-chamber views. The global RVGLS was obtained in the 4-chamber view [15, 16].

### Reproducibility

To determine the reproducibility of the myocardial strain measurements, LA, LV and RV global deformation parameters in 25 random cases (9 T2DM patients, 9 T2DM-HT patients and 7 normal controls) were measured twice at 2-week intervals by a radiologist. Then, another independent investigator who was blinded to the first investigator’s measurements again measured the same images from the 25 randomly selected individuals. Finally, based on the results of the two investigators, the interobserver variability was evaluated.

### Statistical analysis

All data were statistically analyzed using standard statistical software (SPSS 21.0 for Windows, IBM, Chicago, IL, USA). The Kolmogorov–Smirnov test was used to check the normality of all continuous data. Normally distributed data and categorical variables are presented as the means ± standard deviations and frequencies (percentages). Differences in continuous variables between two groups were compared using an independent-sample Student’s t test, and the chi-square test was used to test for differences between categorical variables. Analysis of variance (ANOVA) was used to assess the differences among the three groups. After adjusting for BMI, SBP, DBP or treatment with medication, atrial-ventricular strain and other myocardial function measures were compared using analysis of covariance (ANCOVA). Pearson’s correlation analysis was used for normally distributed variables. Multiple linear regression analyses were used to identify determinants of LA strain in T2DM patients with and without hypertension.

## Results

We recruited subjects (52 patients and 32 controls) for this study and acquired CMR imaging data for all subjects. However, the data from one subject had to be excluded due to poor image quality (severe motion artifacts in one control), and data from two patients were excluded because of abnormal MRI findings (abnormal wall motion in two T2DM-HT patients). Thus, the final study cohort comprised 27 T2DM patients, 23 T2DM-HT patients and 31 healthy controls. The general characteristics of the study subjects are summarized in Table 1. The T2DM-HT group had significantly greater SBP and DBP than the control group (131.7 ± 9.8 vs. 123.9 ± 9.1, *p* < 0.001; 84.2 ± 8.6 vs. 74.9 ± 8.0; *p* < 0.001, respectively). No significant differences in HbA1c, microalbuminuria (MA), medications, serum glucose, fasting blood samples, diabetes duration, creatinine, TG, TC, BUN, HDL-C or LDL-C were observed.

**Table 1:** Clinical characteristics of study subjects

	T2DM (*n* = 27)	T2DM-HT (*n* = 23)	Control (*n* = 31)	*P* value
Age (years)	54.1 ± 7.5	56.8 ± 6.5	54.1 ± 6.2	0.271
Male, *n* (%)	16(59.3)	12(52.2)	16(51.6)	0.819
BMI (kg/m^2^)	25.2 ± 3.4	26.1 ± 2.8^$^	24.2 ± 2.0	0.042
Diabetes duration (y)	8.0 ± 2.8	8.7 ± 3.1	–	0.673
Duration of hypertension (y)	–	4.8 ± 1.2	–	–
SBP (mmHg)	122.5 ± 7.9	131.7 ± 9.8^$^	123.9 ± 9.1	<0.001
DBP (mmHg)	73.9 ± 6.7	84.2 ± 8.6^$^	74.9 ± 8.0	<0.001
BUN (mmol/L)	5.2 ± 1.2	5.4 ± 1.9	–	0.059
creatinine (μmol/L)	69.0 ± 15.3	68.8 ± 14.2	–	0.527
Total cholesterol (mmol/L)	4.3 ± 0.9	4.5 ± 1.0	–	0.630
Triglycerides (mmol/L)	1.6 ± 1.5	1.6 ± 0.9	–	0.263
HDL-C (mmol/L)	1.3 ± 0.3	1.2 ± 0.3	–	0.986
LDL-C (mmol/L)	2.4 ± 0.7	2.7 ± 0.7	–	0.575
FPG (mmol/L)	8.1 ± 3.6	8.5 ± 2.9	–	0.596
Hemoglobin A1C (%)	9.4 ± 2.4	8.3 ± 2.2	–	0.639
Microalbuminuria (MA)	11.5 ± 6.3	9.0 ± 6.3	–	0.483
Diabetic complication, *n* (%)				
Retinopathy	7(25.9)	4(17.4)	–	0.353
Neuropathy	5(18.5)	3(13)	–	0.448
Peripheral vascular disease	4(14.8)	6(26.1)	–	0.261
Hypoglycemic medication, *n* (%)				
Insulin	13(48.1)	10(43.5)	–	0.482
Metformin	16(59.3)	13(56.5)	–	0.569
Sulphonylurea	4(14.8)	6(26.1)	–	0.261
Other medication, *n* (%)				
Statin	8(29.6)	6(26.1)	–	0.517
Aspirin	7(25.9)	10(43.5)	–	0.157
Antihypertensive medication, *n* (%)				
ACEI	–	11(47.8)	–	–
Diuretics	–	8 (34.7)	–	–
Calcium channel blockers	–	3 (13.0)	–	–
β-blockers	–	4 (17.4)	–	–

All data expressed as mean ± SD, percentage (number of participants), or median (interquartile range), as appropriate. $ significant difference between T2DM-HT patients and control groups, p<0.05

T2DM-HT, type 2 diabetes mellitus-hypertension; BMI, body mass index; HR, heart rate; SBP, systolic blood pressure; DBP, diastolic blood pressure; BUN, blood urea nitrogen; HDL-C, high-density lipoprotein cholesterol; LDL-C, low-density lipoprotein cholesterol; FPG, fasting plasma glucose; ACEI, angiotensin-converting enzyme inhibitor

Table 2 shows comparisons of various parameters of MRI characteristics among subjects. LV myocardial strain was not significantly different among the three groups. LAGCS was significantly greater in the T2DM-HT group than in the control group (LAGCS: 39.4 ± 12.7% vs 33.9 ± 8.7%, *p* < 0.05). LAGCS, LAGRS and LAGLS were significantly lower in the T2DM group than in the control group (LAGCS: 27.6 ± 3.6% vs 33.9 ± 8.7%; LAGRS: − 29.2 ± 4.7% vs − 32.9 ± 3.9%; LAGLS: 23.8 ± 5.5% vs 30.9 ± 6.0, *p* < 0.05 for all). LAGCS, LAGRS and LAGLS were significantly greater in the T2DM-HT group than in the T2DM group (LAGCS: 39.4 ± 12.7% vs 27.6 ± 3.6%; LAGRS: − 34.8 ± 7.3% vs − 29.2 ± 4.7%; LAGLS: 33.5 ± 6.7% vs 23.8 ± 5.5%, *p* < 0.05 for all). RVGLS was significantly lower in the T2DM and T2DM-HT groups than in the control group (RVGLS: − 22.0 ± 3.4% vs − 26.0 ± 7.4%, − 21.1 ± 5.5% vs − 26.0 ± 7.4%; *p* < 0.05, respectively).

**Table 2:** MRI characteristics of study population

	T2DM (*n* = 27)	T2DM-HT (*n* = 23)	Control (*n* = 31)	*P* value
LVEDV index (mL/m^2^)	59.8 ± 15.3	62.5 ± 8.5	64.1 ± 11.1	0.404
LVESV index (mL/m^2^)	27.5 ± 5.7	29.1 ± 7.8	27.7 ± 6.3	0.681
SV	34.5 ± 6.2	33.4 ± 2.9	36.4 ± 6.0	0.131
LVEF (%)	56.2 ± 5.3	58.8 ± 4.5	57.2 ± 4.2	0.143
LVM index (g/m^2^)	56.6 ± 8.6	57.9 ± 5.5	55.5 ± 6.4	0.473
LAV index (mL/m^2^)	37.9 ± 7.8	36.4 ± 10.1	37.1 ± 11.1	0.866
LVGRS (%)	87.9 ± 27.6	80.4 ± 32.2	78.7 ± 18.6	0.382
LVGCS (%)	−21.2 ± 4.4	−20.1 ± 3.9	−21.4 ± 2.6	0.364
LVGLS (%)	−22.5 ± 3.6	−22.0 ± 4.5	− 21.9 ± 2.7	0.815
LAGRS (%)	−29.2 ± 4.7^*#^	−34.8 ± 7.3	−32.9 ± 3.9	0.001
LAGCS (%)	27.6 ± 3.6^*#^	39.4 ± 12.7^$^	33.9 ± 8.7	<0.001
LAGLS (%)	23.8 ± 5.5^*#^	33.5 ± 6.7	30.9 ± 6.0	<0.001
RVGLS (%)	−22.0 ± 3.4^#^	−21.1 ± 5.5^$^	−26.0 ± 7.4	0.005

* significant difference between T2DM patients and T2DM-HT patients, p<0.05; # significant difference between T2DM patients and control groups, p<0.05; $ significant difference between T2DM-HT patients and control groups, p<0.05

T2DM-HT, type 2 diabetes mellitus with hypertension; LVEDV, left ventricular end-diastolic volume; LVESV, left ventricular end-systolic volume; SV: stroke volume; LVEF, left ventricular ejection fraction; LVM, left ventricular mass; LAV, left atrial volume; LVGRS, left ventricular global radial strain; LVGCS, left ventricular global circumferential strain; LVGLS, left ventricular

**Fig. 2:**
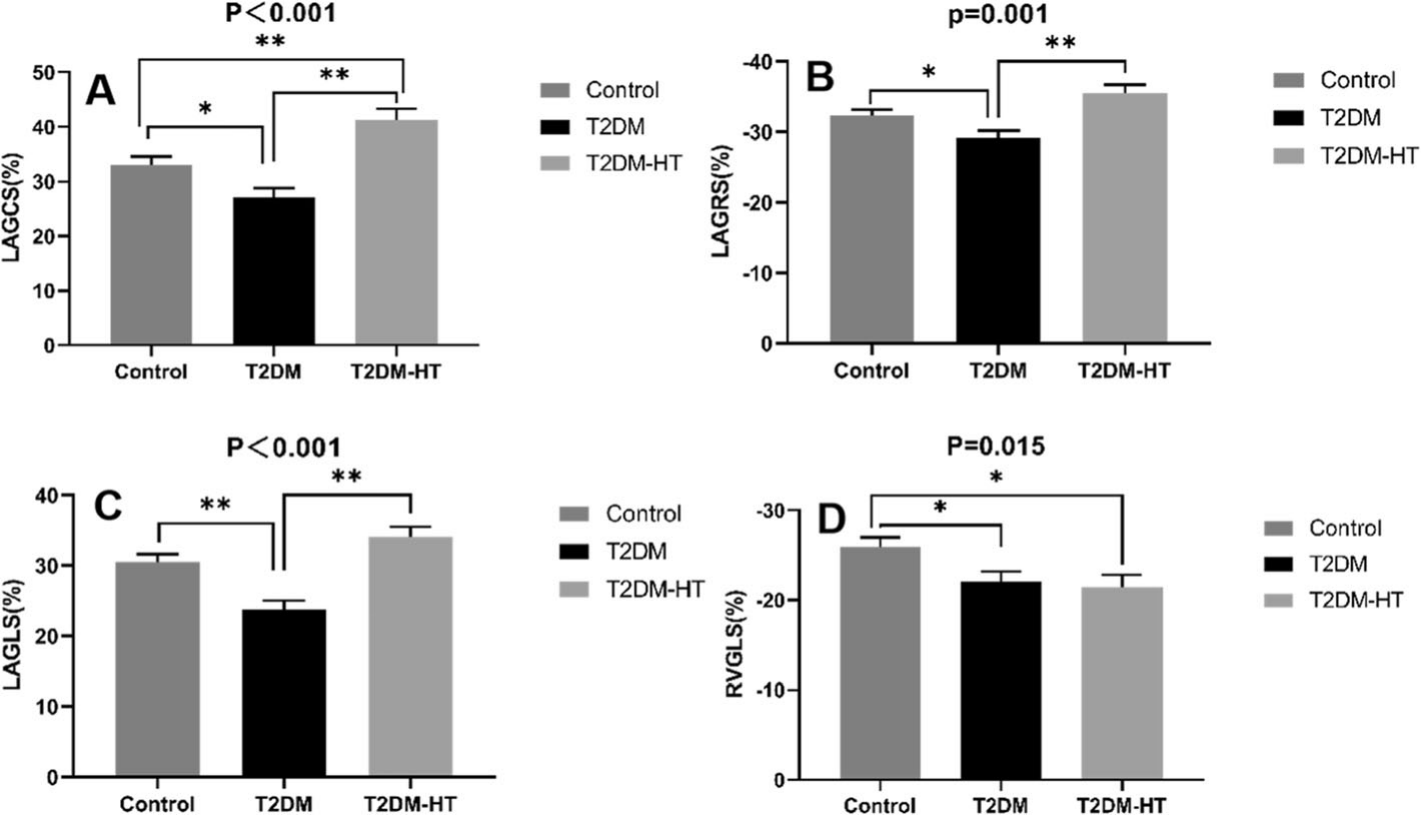
Comparison of LAGCS (**a**), LAGRS (**b**), LAGLS (**c**) and RVGLS (**d**) values among the healthy controls, T2DM and T2DM-HT group. RVGLS, right ventricle global longitudinal strain. LAGCS, left atrial global circumferential strain (GCS); T2DM, type 2 diabetes mellitus; T2DM-HT, type 2 diabetes mellitus with hypertension

**Fig. 3:**
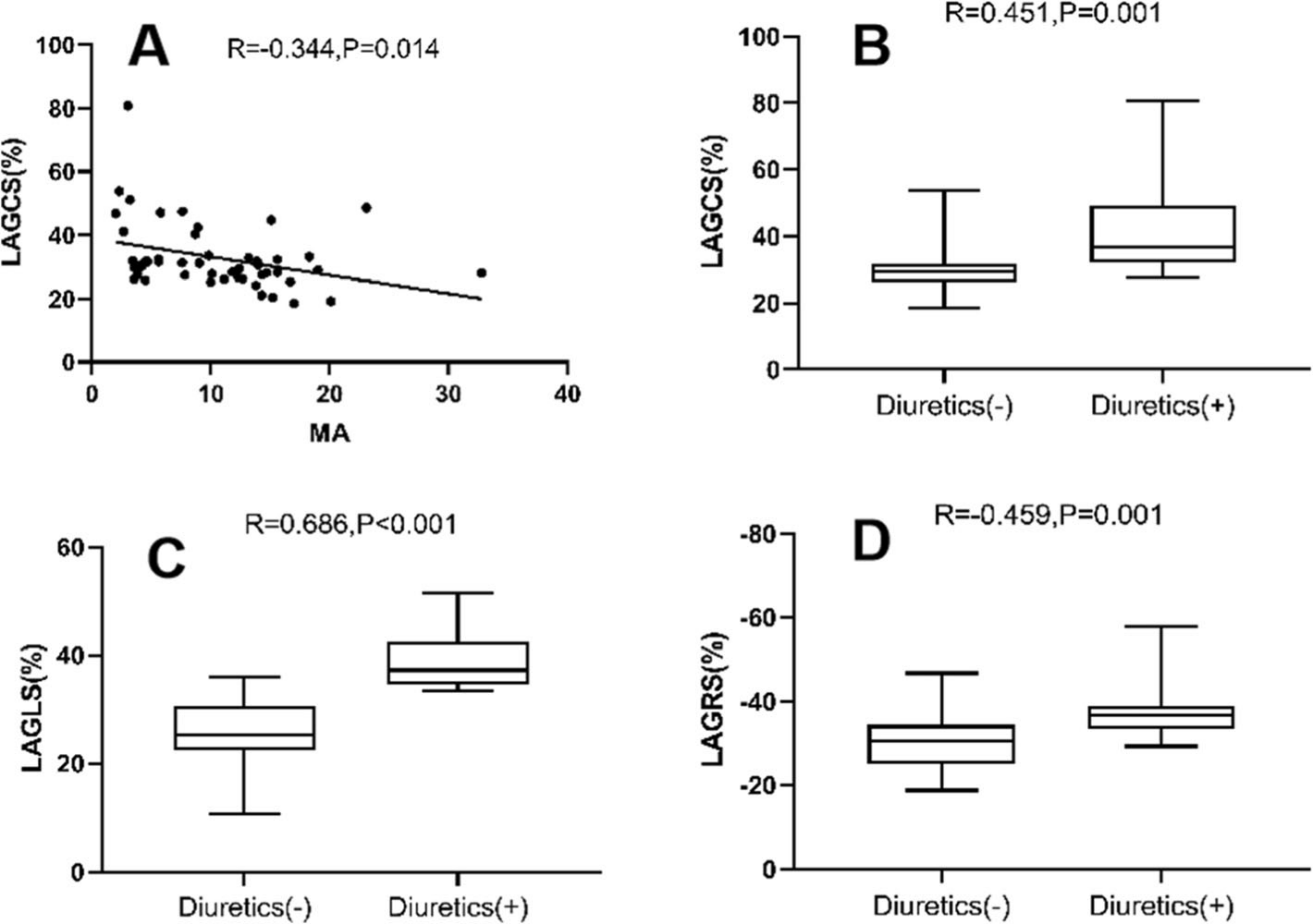
The relationship between LAGCS value and MA level in diabetic patients (**a**); The relationship between LAGCS value and diuretic treatment in diabetic patients (**b**); The relationship between LAGLS value and diuretic treatment in diabetic patients (**c**); The relationship between LAGRS value and diuretic treatment in diabetic patients (**d**); LAGCS, left atrial global circumferential strain; MA, microalbuminuria; LAGLS, left atrial global longitudinal strain; LAGRS, left atrial global radial strain

Table 3 shows comparisons of various parameters of MRI characteristics among subjects; the comparisons are adjusted for BMI, SBP, and DBP. LV myocardial strain was not significantly different among the three groups. LAGCS was significantly greater in the T2DM-HT group than in the control group (LAGCS: 41.2 ± 2.1% vs 33.0 ± 1.6%; *p* < 0.001) (Fig. 2a). LAGCS, LAGRS and LAGLS were significantly lower in the T2DM group than in the control group (LAGCS: 27.1 ± 1.7% vs. 33.0 ± 1.6%; LAGRS: − 29. 2 ± 1.0% vs. -32.3 ± 0.9%; LAGLS: 23.8 ± 1.2% vs. 30.5 ± 1.1%; *p* < 0.05 for all). LAGCS, LAGRS and LAGLS were significantly higher in the T2DM-HT group than in the T2DM group (LAGCS: 41.2 ± 2.1% vs. 27.1 ± 1.7%; LAGRS: − 35.5 ± 1.2% vs. -29.2 ± 1.0%; LAGLS: 34.1 ± 1.4% vs. 23.8 ± 1.2%, *p* < 0.05 for all) (Fig. 2a-c). RVGLS was significantly lower in the T2DM and T2DM-HT groups than in the control group (RVGLS: − 22.0 ± 1.2% vs. -25.9 ± 1.1%, − 21.4 ± 1.4% vs. -25.9 ± 1.1%, *p* < 0.05, respectively) (Fig. 2d). In the diabetic patients, LAGCS showed a significant negative correlation with MA levels (*r* = − 0.344, *p* = 0.014) (Table 4) (Fig. 3a). The improvement of LAGCS, LAGLS and LAGRS might be associated with diuretic treatment (*r* = 0.451, *p* = 0.001; *r* = 0.686, *p* < 0.001; *r* = − 0.459, *p* = 0.001, respectively) (Table 4) (Fig. 3b-d).

**Table 3:** MRI characteristics of study population adjusted for BMI, SBP, DBP

	T2DM (*n* = 27)	T2DM-HT (*n* = 23)	Control (*n* = 31)	*P* value
LVEDV index (mL/m^2^)	59.3 ± 2.5	63.3 ± 2.9	64.0 ± 2.3	0.336
LVESV index (mL/m^2^)	27.5 ± 1.3	29.5 ± 1.6	27.5 ± 1.2	0.599
SV	34.4 ± 1.1	34.0 ± 1.3	36.1 ± 1.0	0.341
LVEF (%)	55.9 ± 1.0	59.2 ± 1.1	57.2 ± 0.9	0.114
LVM index (g/m^2^)	56.5 ± 1.4	58.0 ± 1.7	55.6 ± 1.3	0.559
LAV index (mL/m^2^)	38.1 ± 2.0	35.6 ± 2.4	37.5 ± 1.8	0.728
LVGRS (%)	90.0 ± 5.2	77.4 ± 6.2	79.0 ± 4.9	0.212
LVGCS (%)	−21.5 ± 0.7	−19.8 ± 0.9	− 21.4 ± 0.7	0.310
LVGLS (%)	−22.7 ± 0.7	−21.5 ± 0.9	−22.1 ± 0.7	0.607
LAGRS (%)	−29.2 ± 1.0^*#^	−35.5 ± 1.2	−32.3 ± 0.9	0.001
LAGCS (%)	27.1 ± 1.7^*#^	41.2 ± 2.1^$^	33.0 ± 1.6	<0.001
LAGLS (%)	23.8 ± 1.2^*#^	34.1 ± 1.4	30.5 ± 1.1	<0.001
RVGLS (%)	−22.0 ± 1.2^#^	−21.4 ± 1.4^$^	−25.9 ± 1.1	0.015

* significant difference between T2DM patients and T2DM-HT patients, p<0.05; # significant difference between T2DM patients and control groups, p<0.05; $ significant difference between T2DM-HT patients and control groups, p<0.05

T2DM-HT, type 2 diabetes mellitus with hypertension; LVEDV, left ventricular end-diastolic volume; LVESV, left ventricular end-systolic volume; SV, stroke volume; LVEF, left ventricular ejection fraction; LVM, left ventricular mass; LAV, left atrial volume; LVGRS, left ventricular global radial strain; LVGCS, left ventricular global circumferential strain; LVGLS, left ventricular global longitudinal strain; LAGRS, left atrial global radial strain; LAGCS, left atrial global circumferential strain; LAGLS, left atrial global longitudinal strain; RVGLS, right ventricular global longitudinal strain

**Table 4:** Univariate regression analysis for LAGLS, LAGCS, LAGRS and RVGLS in normotensive or hypertensive T2DM patients

	LAGLS (%)		LAGCS (%)		LAGRS (%)		RVGLS(%)	
Variable	*R* value	*P* value	*R* value	*P* value	*R* value	*P* value	*R* value	*P* value
Age (years)	−0.019	0.897	−0.047	0.745	0.219	0.126	−0.094	0.516
sex	0.003	0.981	− 0.151	0.294	0.205	0.153	0.009	0.953
BMI (kg/m^2^)	−0.086	0.553	−0.154	0.285	0.272	0.056	0.033	0.821
SBP (mmHg)	0.134	0.353	0.103	0.475	−0.035	0.809	0.221	0.123
DBP (mmHg)	0.181	0.209	0.219	0.127	−0.155	0.282	−0.001	0.997
Diabetes duration (y)	0.056	0.697	0.025	0.861	0.136	0.347	−0.214	0.136
BUN (mmol/L)	−0.084	0.562	−0.058	0.687	0.169	0.241	−0.276	0.052
Creatinine (μmol/L)	−0.016	0.914	−0.107	0.460	0.243	0.090	−0.093	0.519
Total cholesterol (mmol/L)	0.030	0.835	−0.029	0.842	0.056	0.699	−0.251	0.079
Triglycerides (mmol/L)	0.095	0.512	0.059	0.682	−0.090	0.534	−0.084	0.564
HDL-C (mmol/L)	−0.020	0.891	−0.252	0.078	0.162	0.261	0.013	0.929
LDL-C (mmol/L)	0.029	0.843	0.021	0.887	0.059	0.684	−0.222	0.122
FPG (mmol/L)	−0.037	0.801	0.127	0.378	−0.087	0.549	−0.134	0.354
Hemoglobin A1C (%)	−0.288^*^	0.042	0.070	0.629	0.037	0.796	0.050	0.729
MA	−0.198	0.169	−0.344^*^	0.014	0.220	0.125	−0.134	0.354
Insulin	−0.156	0.279	−0.168	0.245	0.123	0.396	−0.120	0.407
Metformin	−0.149	0.301	−0.111	0.442	0.152	0.293	−0.220	0.125
Sulphonylurea	0.034	0.813	0.106	0.465	0.052	0.722	0.003	0.981
Statin	0.048	0.738	0.165	0.252	−0.110	0.446	−0.117	0.418
Aspirin	0.052	0.721	0.222	0.120	−0.054	0.711	−0.021	0.885
ACEI	0.098	0.497	0.065	0.653	0.099	0.493	−0.099	0.493
Diuretics	0.686^**^	0.000	0.451^**^	0.001	−0.459^**^	0.001	0.260	0.068
Calcium channel blockers	0.041	0.780	−0.017	0.907	0.122	0.399	−0.076	0.602
β-blockers	−0.251	0.079	−0.050	0.728	0.277	0.051	0.107	0.462

LAGLS, left atrial global longitudinal strain; LAGCS, left atrial global circumferential strain; LAGRS, left atrial global radial strain; RVGLS, right ventricular global longitudinal strain

BMI, body mass index; HR, heart rate; SBP, systolic blood pressure; DBP, diastolic blood pressure; BUN, blood urea nitrogen; HDL-C, high-density lipoprotein cholesterol; LDL-C, low-density lipoprotein cholesterol; FPG, fasting plasma glucose; MA, microalbuminuria; ACEI, angiotensin-converting enzyme inhibitor

After further adjustment for medication (Table 5), the LV myocardial strain was not significantly different among the three groups. LAGCS was significantly greater in the T2DM-HT group than in the control group (LAGCS: 40.4 ± 3.8% vs 33.5 ± 2.4%, *p* < 0.05). LAGCS, LAGRS and LAGLS were significantly lower in the T2DM group than in the control group (LAGCS: 27.2 ± 2.1% vs 33.5 ± 2.4%; LAGRS: − 28.6 ± 1.1% vs − 31.9 ± 1.3%; LAGLS: 24.3 ± 1.3% vs 31.4 ± 1.5, *p* < 0.05 for all). LAGCS, LAGRS and LAGLS were significantly greater in the T2DM-HT group than in the T2DM group (LAGCS: 40.4 ± 3.8% vs 27.2 ± 2.1%; LAGRS: − 36.8 ± 2.0% vs − 28.6 ± 1.1%; LAGLS: 32.3 ± 2.4% vs 24.3 ± 1.3%, *p* < 0.05 for all). RVGLS was significantly lower in the T2DM and T2DM-HT groups than in the control group (RVGLS: − 21.4 ± 1.2% vs − 26.3 ± 1.1%, − 21.4 ± 1.4% vs − 26.3 ± 1.1%; *p* < 0.05, respectively). In the univariate analysis, the HbA1c level was negatively correlated with LAGLS, but in the multivariate analysis, there was no correlation. In the multivariable stepwise analysis, the independent determinant of LAGLS was diuretic treatment (β = 0.643, *p* < 0.001) (model R2 = 0.464), the independent determinants of LAGCS were MA (β = − 0.289, *p* = 0.021) and diuretic treatment (β =0.440, *p* = 0.001) (model R2 = 0.341), and the independent determinant of LAGRS was diuretics (β = − 0.442, *p* = 0.001) (model R2 = 0.345) (Table 6).

**Table 5:** MRI characteristics of study population adjusted for BMI, SBP, DBP and medicine treatment

	T2DM (*n* = 27)	T2DM-HT (*n* = 23)	Control (*n* = 31)	*P* value
LVEDV index (mL/m^2^)	57.5 ± 3.1	68.4 ± 5.5	61.8 ± 3.5	0.230
LVESV index (mL/m^2^)	26.1 ± 1.6	34.1 ± 2.9	25.3 ± 1.8	0.104
SV	33.5 ± 1.4	35.8 ± 2.5	35.5 ± 1.6	0.512
LVEF (%)	55.7 ± 1.1	57.9 ± 2.0	58.3 ± 1.3	0.223
LVM index (g/m^2^)	56.3 ± 1.7	57.7 ± 3.0	56.0 ± 1.9	0.920
LAV index (mL/m^2^)	39.5 ± 2.3	32.0 ± 4.2	38.9 ± 2.7	0.394
LVGRS (%)	83.1 ± 5.7	84.2 ± 10.1	80.1 ± 6.5	0.926
LVGCS (%)	−20.8 ± 0.8	−20.5 ± 1.4	−21.5 ± 0.9	0.794
LVGLS (%)	−22.0 ± 0.8	−22.9 ± 1.5	−21.6 ± 1.0	0.853
LAGRS (%)	−28.6 ± 1.1^*#^	−36.8 ± 2.0	−31.9 ± 1.3	0.003
LAGCS (%)	27.2 ± 2.1^*#^	40.4 ± 3.8^$^	33.5 ± 2.4	0.008
LAGLS (%)	24.3 ± 1.3^*#^	32.3 ± 2.4	31.4 ± 1.5	<0.001
RVGLS (%)	−21.4 ± 1.2^#^	−21.4 ± 1.4^$^	−26.3 ± 1.1	0.008

* significant difference between T2DM patients and T2DM-HT patients, p<0.05; # significant difference between T2DM patients and control groups, p<0.05; $ significant difference between T2DM-HT patients and control groups, p<0.05

T2DM-HT, type 2 diabetes mellitus-hypertension; LVEDV, left ventricular end-diastolic volume; LVESV, left ventricular end-systolic volume; LVEF, left ventricular ejection fraction; LVM, left ventricular mass; LAV, left atrial volume; LVGRS, left ventricular global radial strain; LVGCS, left ventricular global circumferential strain; LVGLS, left ventricular global longitudinal strain; LAGRS, left atrial global radial strain; LAGCS, left atrial global circumferential strain; LAGLS, left atrial global longitudinal strain; RVGLS, right ventricular global longitudinal strain

**Table 6:** multivariate regression analysis for LAGLS, LAGCS and LAGRS in normotensive or hypertensive T2DM patients

Variable	Unstandardized β	Standardized β	*P* value
LAGLS			
Diuretics	12.368	0.643	<0.001
LAGCS			
MA	−0.486	− 0.289	0.021
Diuretics	11.654	0.440	0.001
LAGRS			
Diuretics	−7.207	−0.442	0.001

LAGLS, left atrial global longitudinal strain; LAGCS, left atrial global circumferential strain; MA, microalbuminuria; LAGRS, left atrial global radial strain

### Intra-observer and inter-observer reproducibility

The intraclass correlation coefficient (ICC) values in the intraobserver analysis were 0.987, 0.810, 0.981, 0.985, 0.923, 0.916 and 0.877 for LVGRS, LVGCS, LVGLS, LAGLS, LAGCS, LAGRS, and RVGLS, respectively. The ICC values in the interobserver analysis were 0.973, 0.706, 0.983, 0.952, 0.955, 0.872 and 0.809 for LVGRS, LVGCS, LVGLS, LAGLS, LAGCS, LAGRS, and RVGLS, respectively.

## Discussion

Our findings suggest that (1) compared to the control group, the T2DM group had significantly deteriorated LA and RV strain, and the amelioration of LA strain in the T2DM-HT group compared with the T2DM group; (2) the MA level was negatively related to the LAGCS value; and (3) the improvement of LAGCS, LAGRS, and LAGLS might be associated with diuretic treatment.

DM is a strong risk factor for atrial fibrillation (AF) rate [17], and likely promotes structural and functional alterations of the LA. Previous studies have indicated that T2DM patients showed a reduction in LA strain indices compared with controls [18, 19], and our study yielded the same finding. There are two possible mechanisms that explain why LA global strain in the T2DM group was significantly lower than that in the control group. First, T2DM can lead to LA fibrosis [20], and a subsequent decrease in LA compliance [21]. Impaired LA compliance results in reduced LA strain [19]. Second, myocardial inflammation occurs in T2DM patients [22] and may cause atrial remodelling [4, 23]. In the T2DM-HT group, the LA strain was significantly greater than that in the T2DM group. One possible explanation for this difference is the effect of hypertension on the myocardium. Hypertension increased LV stiffness, blood flow from the LA into the LV was affected, and LA showed an increase in preload in a certain range. Within certain limits, contraction of the LA also follows the Frank–Starling mechanism, which means that the work of LA contraction depends on the volume before its active contraction preload. Thus, LA deformation may be compensatorily enhanced when the LA preload increases within a certain range [24, 25]. Another possible explanation for this difference is the confounding effect of some antihypertensive treatments used by T2DM patients with coexisting hypertension. In a previous experimental study, renin-angiotensin system (RAS) inhibition effects were found to prevent angiotensin II concentration, phosphorylated ERK expression, caspase-3 activity and increased apoptosis, suggesting a beneficial effect on atrial myocardium [26]. Renin-angiotensin system inhibitors (ACEI) can improve LA strain in patients with hypertension [27]. Furthermore, longitudinal dysfunction might be reversed by diuretic treatment in hypertensive patients [28]. However, a prior study [18] indicated that the coexistence of T2DM and hypertension further depressed LA strain in an additive way. The above diferences in the LA strain measurements may be due to diferences in the study populations and diferent strain acquisition methods. Specifcally, the mean age was 64.7 years in T2DM-HT patients in Mondillo’s study, whereas the mean age in our T2DM-HT patients was relatively young, approximately 56.8 years. The literature reports increasing age is independently associated with deteriorated left atrial systolic strain [29]. Second, our strain acquisition method was MR-derived tissue tracking technology, whereas Mondillo’s study employed ultrasound speckle tracking. However, to our knowledge, there are few studies on LA strain changes in T2DM patients, especially those with coexisting hypertension. Whether amelioration of LA strain in T2DM-HT patients can be ascribed to the true effect of HT or to a confounding effect of some antihypertensive treatment still requires further study.

In our study, CMR-derived LV strains were similar among the three groups regardless of the presence of coexisting hypertension. We did not observe that a significantly decreased GLS in T2DM patients compared with normal subjects [30]. The above difference may lie in the duration of diabetes in the study populations. Specifically, the mean duration of diabetes in the longer-term T2DM group was approximately 11 years in Liu’s study [30], whereas the duration in our study was relatively short at only approximately 8 years. In this study, we also found that the LA strain changed significantly, indicating that LA deformation-related impairment could appear even earlier than the LV strain in the early stages of DCM, a finding that is consistent with Cameli’s study [31]. This finding revealed that LV strain did not represent the most accurate parameter for detecting early damage in those patients. A possible explanation may be related to anatomy: the LA is a very thin, single-layer wall that is very sensitive to even subtle stimuli [31]. Therefore, LA strain, as an early parameter, may have been more sensitive than LV strain for the detection of early DCM in our study.

Recently, RV function has received increasing attention and has been deemed to be clinically and prognostically significant in various diseases [32, 33]. RV impairment might be a component of DCM [34, 35]. The present study also confirmed this point: RVGLS was significantly reduced in the T2DM group compared with the control group, which is in agreement with Ng’s study [36]. One possible mechanism is that myocardial triglyceride content is increased in T2DM [37], and the intracellular surplus of triglyceride itself is likely to contribute to myocardial steatosis, which may contribute to significantly greater impairment of RVGLS [36]. Furthermore, a recent animal study also indicated that therapeutic interventions aimed at reducing myocardial triglyceride accumulation have shown beneficial myocardial effects [38]. When coexisting hypertension was present, no significant difference in the RV strain parameter was observed. Recently, Hwang et al. also showed that cardiovascular risk factors, such as hypertension, and RV strain parameter values were similar between individuals with risk factors and those without risk factors [39]. A possible explanation is that T2DM is likely related to subclinical RV systolic and diastolic dysfunction, regardless of coexisting hypertension [40].

The recommended treatment for diabetes is usually a combination of drugs [41], which may include diuretics. Our findings demonstrated that diuretic treatment was significantly related to greater LA global strain. A previous study demonstrated that longitudinal dysfunction might be reversed by diuretic treatment in hypertensive patients [28]. These results and the results of our study suggest that diuretic treatment exerts a protective effect on or reverses LA myocardial strain. Nonetheless, whether diuretics can prevent, delay, or reverse the impairment of LA strain requires further study, and additional information is needed to determine whether there are correlations among the duration of diuretic treatment, the order of diuretic treatment and T2DM diagnosis, and LA strain values. Furthermore, it is well known that DCM is associated with diabetic complications, such as diabetic nephropathy. Previous studies have supported an association between myocardial dysfunction and diabetic nephropathy [42]. In our study, we found that the LAGCS value was correlated with MA level in T2DM. Similarly, Jensen et al. [43] also demonstrated that the degeneration of systolic myocardial function is mainly related to the presence of MA in T1DM. A previous study noted [44] that circumferential strain is mainly generated by subepicardial myofiber contraction. Thus, LAGCS increased after the MA level decreased, which means that LA subepicardial myofiber contraction improved. Thus, the reduction of MA levels is necessary for diabetic patients. MA has traditionally been identified as the earliest marker of diabetic nephropathy. The presence of MA indicates the occurrence of proteinuria, which plays a key role in the progression of early renal dysfunction to end-stage renal disease [45]. However, MA may be temporary and does not always represent permanent kidney damage [46]. Stehouwer’s [47] study showed that diuretics can decrease albuminuria, which may provide effective theoretical guidance for clinical intervention in the treatment of diabetic complications.

In this study, several limitations should be considered. First, our sample size was relatively small, which greatly reduced the power of the study and did not allow us to draw generalized conclusions. We will continue to recruit participants and expand our sample size in our future studies on this topic. Second, several software programs can be used to analyze LA and biventricular deformation, and data on LA and biventricular strain quantification using CMR-FT are insufficient. Thus, reference values for those strains should be determined. Third, other biochemical indices (renal function, microalbuminuria, cholesterol, fasting plasma glucose and hemoglobin A1C levels, etc.) of the controls were not measured at the time of CMR. However, we obtained a detailed medical history for the controls and checked their medical examination reports within 6 months of enrollment to guarantee that our controls met the inclusion criteria. Fourth, the lack of sufficient prospective trials validating the use of left trial indices, such as LAGLS, in evaluating patients for cardiomyopathy is very limiting. Therefore, the use of LA indices to evaluate patients for cardiomyopathy requires further study. Fifth, in our study, CMR-FT was applied to cine SSFP sequences featuring 25 phases per cardiac cycle. Therefore, the temporal resolution was lower than that of speckle tracking echocardiography, and this difference is likely to be relevant, especially in the use of strain to evaluate diastolic function.

## Conclusions

T2DM patients with preserved LV function demonstrated impaired LAGRS, LAGLS, LAGCS and RVGLS compared with controls. The amelioration of LA strain might be associated with hypertensive compensation or antihypertensive treatment. LA strain impairment may appear even earlier than LV myocardial strain in the early stage of DCM. Future follow-up studies are needed to assess the potential prognostic significance of LA and biventricular deformation in diabetic and hypertensive patients.

## Data Availability

The datasets used and analyzed during the current study are available from the corresponding author on reasonable request.

## Abbreviations

CVD: Cardiovascular disease
DCM: Diabetic cardiomyopathy
CAD: Coronary artery disease
HT: Hypertension
LA: Left atrial
RV: Right ventricle
LV: Left ventricle
T2DM: Type 2 diabetes mellitus
T2DM-HT: Type 2 diabetes mellitus with hypertension
CMR-FT: Cardiovascular magnetic resonance feature tracking
LVEDV: Left ventricular end-diastolic volume
LVESV: Left ventricular end-systolic volume
SV: Stroke volume
LVEF: Left ventricular ejection fraction
LVM: Left ventricular mass
LAV: Left atrial volume
LVGRS: Left ventricular global radial strain
LVGCS: Left ventricular global circumferential strain
LVGLS: Left ventricular global longitudinal strain
LAGRS: Left atrial global radial strain
LAGCS: Left atrial global circumferential strain
LAGLS: Left atrial global longitudinal strain
RVGLS: Right ventricular global longitudinal strain
HbA1c: Hemoglobin A1c
MA: Microalbuminuria
TG: Triglycerides
TC: Total cholesterol
BUN: Urea nitrogen
HDL-C: High-density lipoprotein cholesterol
LDL-C: Low-density lipoprotein cholesterol
ACEI: Angiotensin-converting enzyme inhibitor
RAS: Renin-angiotensin system
ICC: Intra-class correlation coefficient
